# A Modification of Periacetabular Osteotomy Using a Two-Incision Approach

**DOI:** 10.2174/1874325000701010013

**Published:** 2007-12-06

**Authors:** Peter Bernstein, Falk Thielemann, Klaus-Peter Günther

**Affiliations:** Department of Orthopaedic Surgery, University Hospital Carl Gustav Carus Dresden, Medical Faculty of the Technical University Dresden, Fetscherstr. 74, D-01307 Dresden, Germany

## Abstract

In residual hip dysplasia periacetabular osteotomy (PAO) can improve insufficient coverage of the femoral head. It requires a broad dissection of the pelvic bones and detachment of muscle insertions, however. We have developed a modification of the Bernese periacetabular osteotomy with reduced soft tissue exposure. It uses two small skin incisions and offers therefore the perspective of nicer scars but also increases the risk of technical complications due to impaired vision. To be able to draft these risks, the clinical and radiographic results of 23 patients with PAO through the modified Smith-Petersen approach of Ganz (group A) and 24 patients with our two-incision modification (group B) have been reviewed retrospectively with an average follow-up of 19 (group A) and 12 (group B) months postoperatively.

Functional improvement (Harris Hip Score) and center-edge-angle normalization did not differ significantly in both groups. Scars of patients in group B were significantly shorter. However, the overall patient satisfaction (as measured with a visual analogous scale) was the same in both groups. 4 patients in group A and one patient in group B developed superficial or deep wound infections.

In conclusion, the experience with our cohort study showed that approach-related morbidity can be reduced without increasing the risk for the individual patient. This observation clearly holds a promise for further minimal invasive approaches as well as for further morbidity reduction of PAO.

Level of Evidence: Retrospective comparative study (Level III).

## INTRODUCTION

To avoid premature development of osteoarthritis in patients suffering from acetabular dysplasia of the hip, reorientation of the dysplastic acetabulum might be necessary. While in children the correction can be achieved over a rather small incision with Salter’s or Pemberton’s osteotomy, it becomes more demanding when treating adolescents and adults. For this age group several approaches to restore a physiological acetabular orientation have been described [1, 2]. The triple osteotomy of Tönnis and the periacetabular osteotomy (PAO) of Ganz are best known and commonly used procedures [1, 3-6]. They allow juxtaarticular mobilization of the acetabular fragment with the possibility to correct the deformity without compromising pelvic symmetry.

While in major corrections Tönnis’ approach generates larger defects between the fragment and the os ischii – therefore increasing the risk of pseudarthrosis, the Bernese osteotomy of Ganz leaves the posterior column intact, requiring less stabilization, preserving blood supply and allowing immediate crutch walking [1, 5, 7]. From the described surgical approaches for periacetabular osteotomy (modified Smith-Petersen, ilio-inguinal, direct anterior) the modified Smith-Petersen approach is considered to be the safest in respect to vessel and nerve protection [8, 9]. A major disadvantage of this approach, however, is the need for extensive exposure and soft tissue release, which might impair functional results and can lead to sometimes visually compromising scars [9]. The latter is even more critical, as most of the patients are young women.

This problem had been addressed by different groups and a two-incision – modification (combined anterolateral and extraperitoneal approach) as well as an endoscopic method had been developed [10, 11]. The results being quite similar to those reported in the literature for PAO, no benefit from the developed approach could be concluded due to missing control groups with a comparable conventional approach. In addition, the endoscopic method revealed to be technically demanding, requiring special equipment and a steep learning curve of the specialized surgeon while resulting in a longer operation time [11].

We have developed another modification of the Bernese periacetabular osteotomy as a first starting point for a stepwise reduction of approach-related morbidity by reducing incision length and limiting muscular release. In our study we wanted to know, if the - so-called - two-incision Smith Peterson approach is as safe and achieves the same morphological correction as the modified Smith Peterson approach of Ganz. In addition we evaluated cosmetic and functional results in order to find out, if there is a difference between the two-incision and the conventional approach. Another aim of our study was to outline the possibilities of further morbidity reduction with the ultimate goal of a stepwise-achieved, safe and optimized periacetabular procedure.

## OPERATIVE TECHNIQUE

The patient is positioned supine on the operating table with the pelvis lying over the radiolucent area. In our patient group A a Bernese periacetabular osteotomy is performed as described through a conventional Smith-Petersen approach [1].

**Fig. (1):**
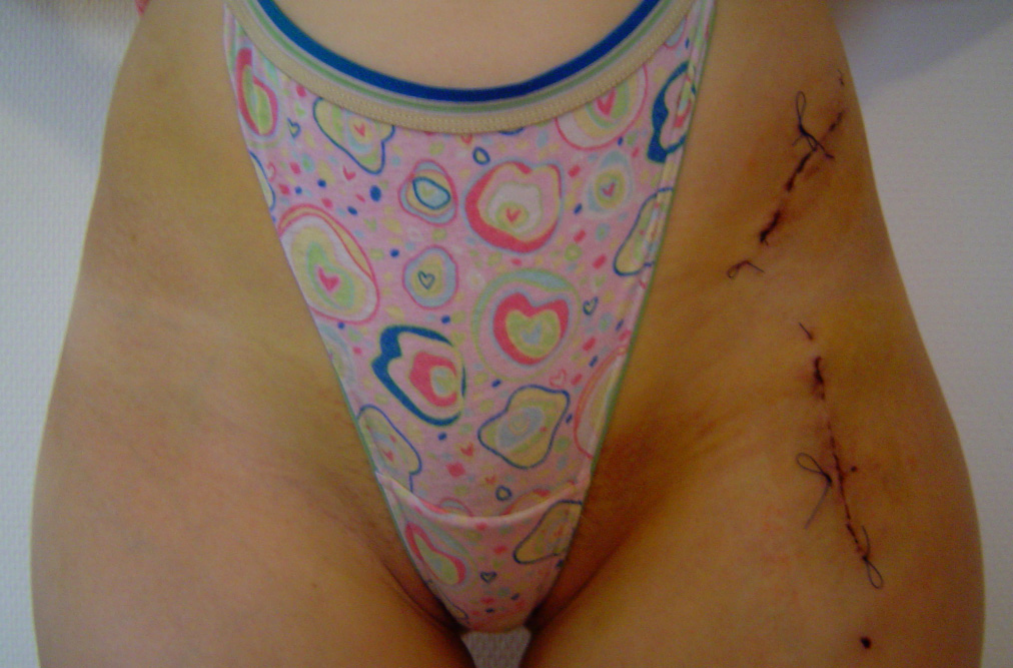
The two-incisions.

In patient group B (two-incision exposure) the Smith-Petersen approach is divided into a proximal cut above the anterior iliac crest and a distal cut over the hip joint (Fig. **1**). Each cut is initially about 7 cm of length and can later be extended (depending on patient anatomy). A bridge of intact skin of at least 5 cm is left in between both incisions. We are beginning with the distal cut, which starts approximately 3 to 4 cm distally of the iliac spine to allow adequate exposure of the femoral neck, pubis and ischium. The superficial aponeurosis of the tensor fasciae latae is incised and blunt dissection separates the muscle from the sartorius fascia, which is left intact to protect the lateral cutaneous femoral nerve. After identification of the rectus femoris origin at the inferior iliac spine the deep fibres of this muscle as well as the fibres of the iliacus muscle are separated from the capsule with a rasp. The rectus femoris muscle tendon is not detached from its pelvic origin. A curved pointed retractor is placed under both muscles onto the anterior wall of the acetabulum. Pubis and ischium are exposed subperiosteally with a long rasp and the hip in 45 degrees of flexion. Blunt Hohman retractors are placed along the inferior and superior border of the pubis and the oblique osteotomy is performed with an oscillating saw. The ischial osteotomy is performed with a 30°-angled chisel as described by the authors of the original technique [1]. We assure correct chisel placement with image intensification.

The second incision is made over the anterior iliac crest and the inside of the ilium exposed subperiosteally. The origin of sartorius muscle and inguinal ligament is detached from the superior anterior iliac spine together with the periosteum. A very limited subperiosteal exposure of the ilium lateral and proximal to the anterior iliac spine is performed to facilitate the osteotomy [1]. Following conventional ilium osteotomy, the acetabular fragment is mobilized with two Schanz screws. When the correct position is reached under control of an image intensifier, preliminary fixation can be accomplished with four K-wires. The joint capsule is opened from anterior to check the labrum and the head-neck offset. Osteochondroplasty can be performed if there is abutment of the femoral head-neck junction against the newly positioned anterior acetabular rim with hip flexion and internal rotation. After capsule closure the definite fixation of the acetabular fragment is accomplished with one or two canulated cancellous screws and additional cortical screws, which replace the K-wires (Fig. **3**). One suction drain is placed near the osteotomy and the aponeurosis, subcutaneous tissue and skin are closed with running sutures.

A general anaesthesia was administered during all procedures. The anticoagulation regimen included low-molecular weight heparins and compression sockets. Postoperative radiographs of all patients (a.p. pelvis) were obtained prior to discharge. All patients received non-steroidal antiinflammatory drugs (50 mg diclophenac three times a day) over two weeks to prevent heterotopic ossification.

Partial weightbearing with two crutches was encouraged for six weeks. Thereafter weekly increased weightbearing was encouraged for further six weeks until full weightbearing (usually three months postoperatively) was reached.

## MATERIAL AND METHODS

This retrospective study was approved by our institutional review board. A cohort of 23 periacetabular osteotomies, performed through a modified Smith-Peterson approach (group A), was followed by 24 osteotomies with reduced skin incision length and less muscular exposure (group B). The patient population in group A consisted of 15 female patients and 8 male patients. In group B 21 female patients and 3 male patients underwent surgery. The median age of all patients was 23 years and did not show a significant difference in both groups (Table **1**).

**Fig. (2):**
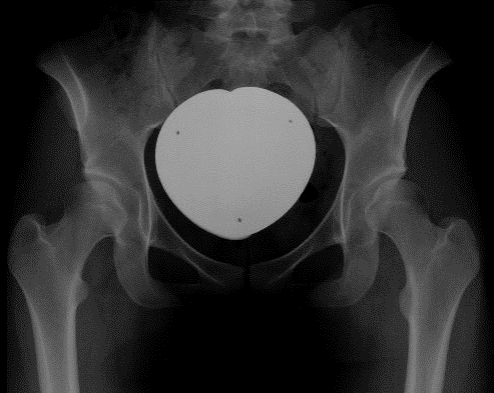
Preoperative radiographic assessment. Indication for surgery included hip pain with the radiological finding of acetabular dysplasia (CE-angle lower than 20 degrees).

**Fig. (3):**
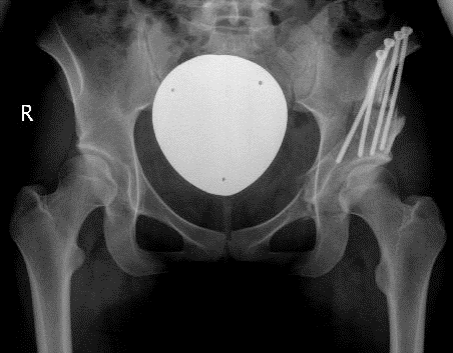
Postoperative radiographic evaluation after two-incision periacetabular osteotomy. Full correction and fixation of the acetabulum was achieved.

Indication for periacetabular osteotomy in both groups was hip pain accompanied with radiologic findings of acetabular dysplasia (radiographically determined center edge angle of less than twenty degrees) in the absence of advanced osteoarthritis (grade three or four according to Kellgren and Lawrence, Fig. **2**). Due to femoroacetabular cam-type impingement, two patients of group A and three patients of group B underwent partial resection of the labrum as well as osteochondroplasty of the femoral head-neck junction in addition to the periacetabular osteotomy. In four patients (group A) and three patients (group B) a proximal femoral osteotomy was performed together with periacetabular osteotomy.

Patients who had a previous pelvic osteotomy or suffered from neuromuscular disease were excluded from the study. All surgeries were performed by the senior author with an experience of more than three-hundred pelvic redirectional osteotomies.

### Clinical Evaluation

As 3 patients were lost to follow-up, 22 patients in each group could be evaluated postoperatively at an average time of 19 months (range 6-29 months) in group A and 12 months (range 6-24) in group B. An orthopaedic resident (first author) performed the clinical evaluation at follow-up.

The Harris hip score was determined preoperatively and at follow-up.

Scars were measured in length and mean width. For the assessment of scar biology the Vancouver Scar Scale (VSS) was used to grade vascularization, pigmentation, thickness and pliability [12]. In addition, patients were asked for signs of discomfort (itching, pain) attributed to the scar. Overall patient satisfaction with the cosmetic appearance of scars was assessed by a Visual Analogous Scale (VAS), ranging from zero (not satisfied at all) to ten (very satisfied).

All complications were recorded and special emphasis was given to sensory nerve lesions. Every patient was tested for signs of hypaesthesia or meralgia. A positive finding was classified as “non-disturbing” when there was a lesion without subjective complaints or classified as “disturbing” if patients felt impaired in any way.

### Radiographic Evaluation

The preoperative and follow-up radiographic examination included an anteroposterior pelvic and false-profile lateral radiograph. Preoperatively a frog-lateral view was obtained as well to assess femoral head-neck offset. On all pre- and postoperative anteroposterior pelvic radiographs the center-edge angle was measured and the presence of osteoarthritis graded according to the criteria of Kellgren and Lawrence [13]. The radiographic evaluation was performed by an orthopaedic resident (first author, non-blinded) with appropriate training.

### Statistical Methods

Statistical analysis was done using a commercially available software package (SPSS™ 12.0G, Apache Software Foundation). All data displayed – if not otherwise stated – is expressed in medians; the minimal and maximal values are reported as range. Calculation of the p-value was done with Wilcoxon’s test and the level of significance was set at p<0.05.

## RESULTS

Surgical process data – as it is summarized in Tables **1** and **2** – shows no significant differences in the performance of the two-incision and the conventional method.

### Complications

Two patients suffered from postoperative complications in group A: In a 18-year old female with poor primary wound healing, a subcutaneous abscess had to be drained six months postoperatively. In a 34-year old male a subacute infection of the hip joint developed 4 months after periacetabular osteotomy. Under antibiotic therapy the infection disappeared, but continuous pain with weightbearing and rapidly progressive joint space narrowing required total joint replacement one year after the periacetabular osteotomy. Further healing was uneventful.

Two other patients in group A and one patient in group B had prolonged wound healing without requiring surgery.

No single patient showed the clinical signs of motor function impairment.

In both groups the rate of sensory abnormalities on neurologic examination was relatively high. In a total of 20 patients (45.5%) hypaesthesia of the proximal lateral thigh indicative of lateral femoral cutaneous nerve lesion could be found. The majority of patients did not feel impaired by that finding, as only one patient in group A and four patients in group B complained about hypaesthesia.

### Morphological Correction

Preoperatively, group B patients had worse CE-angles than patients from group A. In both groups similar CE-angles were achieved postoperatively. Therefore we observed a (pseudo-)significantly higher CE-angle improvement in group B (21 degrees) than in group A (17 degrees, p=0.017, Table **3**).

Although in both groups 5 patients showed radiographic nonunion of the pubic at follow-up, no single nonunion was painful or required revision. No nonunion of the ilium or ischium was diagnosed radiographically. The administered prophylaxis against heterotopic ossification was rated safe, despite the possibility of NSAID-suppressed bone healing.

### Functionality

Functionality was neither preoperatively nor postoperatively significantly different between both groups (Table **3**). The Harris hip score improved from 69 (range 52 to 100) preoperatively to 86 (range 52 to 100) postoperatively in group A and from 66 (range 37 to 86) preoperatively to 93 (range 70 to 100) postoperatively in group B. Although the functional improvement as measured by the difference between postoperative and preoperative Harris hip score was higher in group B (22 points; range -3 to 50 points), the difference was not statistically significant.

### Cosmetic Results

**Fig. (4):**
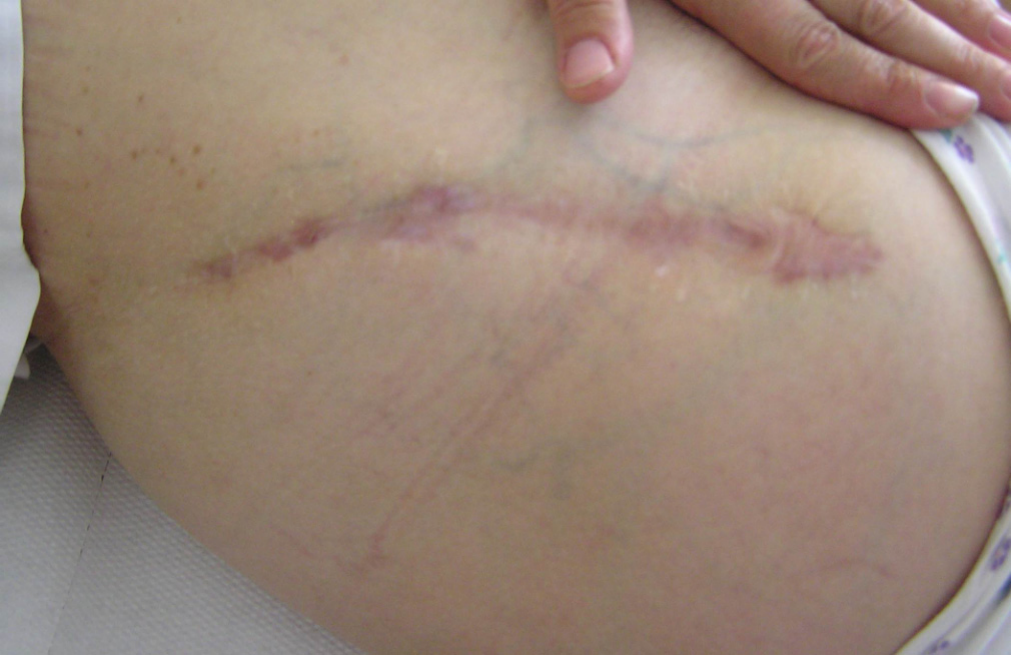
Scar appearance of the conventional modified Smith-Petersen approach.

**Fig. (5):**
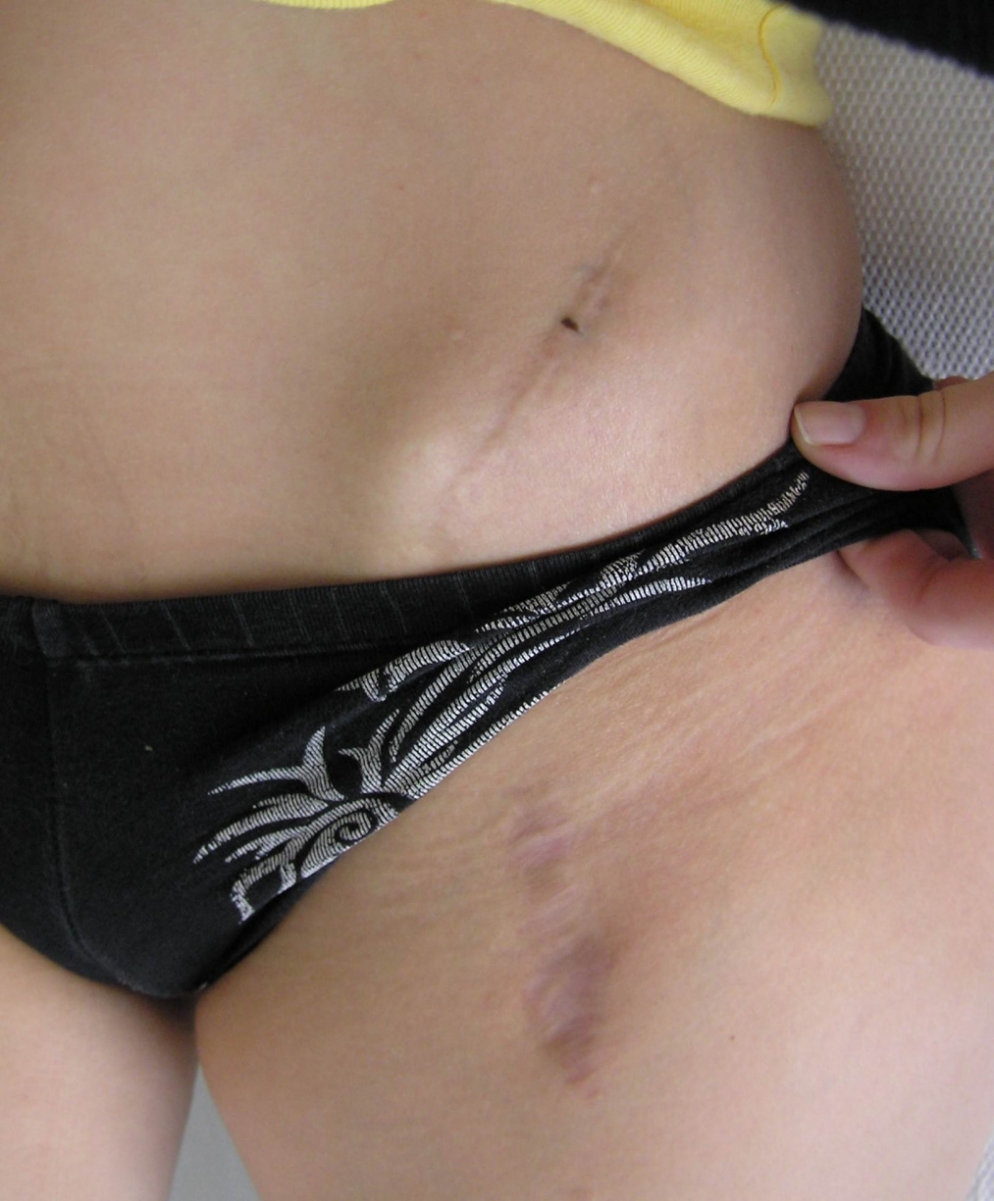
Scar appearance of the two-incision technique.

Average scar length in patients with conventional osteotomy was 21 cm (range 18-26 cm, Fig. **4**). If the proximal and distal incision scars in patients with less-invasive surgery were added, the average length was 18 cm (range 14-23 cm, Fig. **5**) and therefore shorter than the conventional approach (p<0.001, Table **4**). Additionally, scars of the two-incision approach were markedly thinner in range (0.3-1.0 cm *vs* 0.5-2.0 cm), although mean scar width showed no significant difference (p=0.58).

In contrast to the differences in scar metrics, scar biology and VAS rating were almost equal in both patient groups (Table **4**). Four patients (two from either group) were complaining about slight discomfort resulting from the scars. All other patients felt comfortable with their scars.

## DISCUSSION

The commonly used modified Smith-Petersen approach for PAO requires a relatively large skin incision, broad dissection of the pelvic bones and detachment of muscle insertions. The originally recommended delay of full motion exercises [8, 9] and decreasing cosmetic acceptance in mostly female patients encouraged us to undertake a stepwise morbidity reduction. As a first step, our two-incision technique permits a less invasive exposure of the osteotomy sites with less muscle detachment and reduced skin incision length when compared to the conventional technique.

Recently, several other authors have also tried to reduce the morbidity of pelvic osteotomies through less invasive approaches [10, 11, 14]. Published reports have not included control groups in their protocol [10, 11] or even not performed a data analysis at all [14]. This is the first cohort study in a sequential morbidity reduction plan and to our knowledge the only one that compares a less-invasive modification of PAO with the conventional approach.

The results of our study indicate, that the potential of acetabular correction in severe dysplasia is not impaired, when a two-incision modification of periacetabular osteotomy instead of the conventional technique is performed. The achieved CE-angle-improvements were as good as the values known from the literature for periacetabular as well as other re-directional pelvic osteotomies [1-5, 7, 10, 11, 15, 16].

This finding is very important, as limited exposure is known to be potentially associated with the risk of malpositioning as for example in minimal invasive hip replacement surgery [17]. As visualization of the periacetabular area is impaired with the two-incision approach, we use intraoperative fluoroscopy to assure appropriate correction of the fragment.

Initially, we expected our less-invasive technique to require more operation time. However, we only observed a non-significant extension of 8 minutes compared to the conventional approach and are therefore faster than other published less invasive techniques [10, 11].

The incision length as well as the scar width could be significantly reduced with the two-incision approach. Most strikingly, the proximal scar appeared to be very thin, so that in the end only one short scar remained visible for crude observation. The biology of the scars, including vascularization, pigmentation, thickness and pliability rated equal in both groups [12]. Also, no difference in patients’ satisfaction (as rated by VAS) could be observed. This finding contrasts to the observation, that almost all of our patients ask for a two incision approach today, when we offer both possibilities.

We observed a smaller frequency of superficial as well as deep infections in patients with the two-incision approach. This could be attributed to the fact, that a bridge of intact skin between the proximal and distal incision reduces the tension on the wound when compared with the original Smith-Petersen approach. Any delay in wound healing might pose a patient to the risk of deep infection. Due to the small number of patients in our study groups, and the fact, that only two patients did develop deep infections – even if both occurred in the group of patients with conventional osteotomy - we can not establish a correlation between the type of exposure and the incidence of wound infects. Although the incidence of deep infections after pelvic osteotomies seems to be relatively low in the literature [1, 7-9, 15], any procedure which reduces the risk of wound healing problems is favourable.

We did not expect to observe functional differences. Consequently the results of the Harris Hip Score in our study showed no significant differences. However, the observation of non-significant differences can be due to methodological reasons (retrospective design without randomization and small number of patients in both groups) but also to the fact, that we did not change our rehabilitation protocol with the implementation of “less invasive” surgery. Theoretically, reduced muscle detachment would allow the application of faster rehabilitation concepts. Although we allowed full range-of-motion exercise immediately after surgery in both patient groups, the duration of partial-weightbearing (6-12 weeks) was similarly recommended. In our next study we will focus on that part.

We did also not record the postoperative pain intensity (i.e. usage of analgesics) and early postoperative function, which might be different in both groups. We do know from minimal invasive total hip replacement, that the overall function of patients three to six months postoperatively is not anymore different from patients with conventional surgery, although early postoperative limping (up to six weeks) might be reduced [18]. The functional improvement at follow-up in our patients is comparable to the reported values from mid- and long-term studies of conventional and less-invasive pelvic osteotomies [2, 5, 10, 15, 19]. A prospective study with tailored rehabilitation concepts and integration of early postoperative function and pain measures would be necessary to evaluate the effects of a less invasive approach more precisely.

Besides a somewhat higher occurence of superficial and deep wound infections in the control group, the complication rate is not different in our patients after conventional and after two-incision pelvic osteotomy. In both groups a relatively high incidence of lateral femoral cutaneous nerve dysfunction (45.5%) is evident. Although this finding is similar to the rate of nerve dysfunctions as described by Pajarinen and Hirvensalvo [10], it is difficult to discuss the clinical relevance. In most reports about pelvic osteotomies the authors concentrate on radiological and functional results without providing detailed information about the rate of this minor complication. Ganz *et al*. [1], however, describes a “relatively frequent dysaesthesia of the lateral femoral cutaneous nerve”. As most patients do not notice the dysfunction at all, the relevance of this complication might be not too significant.

The absence of motor-nerve- and vessel-lesions validates the safety of the Smith-Petersen approach in the conventional as well as in our less-invasive modification

In conclusion, our retrospective investigation has shown, that a two-incision modification of the periacetabular osteotomy can be performed safely. In spite of the limited approach, the potential for radiographic improvement of femoral head coverage is not different from the Bernese technique. A significant shorter incision length makes the procedure appealing for patients. At follow-up, functional scores and patient satisfaction are similar in both groups.

Although patients were not selected for either group (consecutive cohorts), we had a different gender ratio and a different follow up that might influence the study results. Therefore interpretation is limited.

As a next step we plan to analyze different rehabilitation protocols in favour of a faster mobilization.

## Figures and Tables

**Table 1.** Distribution of Patient Characteristics Between the Groups with Conventional Approach (Group A) and Two-Incision Approach (Group B)

	Total	Group A	Group B
Number of PAOs	47	23	24
Sex (f: m ratio)	3,3: 1	1,9: 1	7: 1
Median Age (range), years	23 (14-46)	27 (16-40)*	20 (14-46)*
Median Follow-up (range), months	16 (6-29)	19 (6-29)	12 (6-24)
Side of PAO, left/right	22/22	8/14	14/8
Preoperative Osteoarthritis	17	10	7

* p=0.495.

**Table 2.** Perioperative Data

	Total	Group A	Group B	p-Value
median operation time (range)	137 (85-295)	133 (85-191)	141 (101-295)	0.12
median operation time (range) excluding additional procedures	130 (85-199)	124 (85-165)	136 (101-199)	0.175
number of patients with additional labral surgery and/or osteo-chondroplasty	5	2	3	
number of patients with additional femoral osteotomy	7	4	3	
number of patients with blood transfusion	11	5	6	
hospitalization	10 (7-16)	11 (7-16)	10 (8-13)	0.167

**Table 3.** Radiological and Functional Results

	Total	Group A	Group B	p-Value
median preoperative CE-angle (range)	12° (0-20)	15° (0-20)	11° (0-18)	0.041
median postoperative CE-angle (range)	30° (18-45)	30° (18-40)	30° (22-45)	0.544
CE-difference (range)	19° (7-44)	17° (7-28)	21° (11-44)	0.017
median preoperative HHS (range)	69 (37-100)	69 (52-100)	66 (37-86)	0.503
median postoperative HHS (range)	88 (52-100)	86 (52-100)	93 (70-100)	0.345
HHS-difference (range)	18 (-17-50)	17 (-17-41)	22 (-3-50)	0.105

Acetabular correction is expressed as CE-difference between post- and preoperative values, functional improvement is represented by the HHS-difference (CE-angle = center-edge angle, HHS= Harris hip score).

**Table 4.** Scar Characteristics (VSS = Vancouver Scar Scale [12], VAS = Visual Analogous Scale)

	Group A	Group B	p-Value
scar length (range)	21 (18-26)	18 (14-23)	0.000
scar width (range)	1.0 (0.5-2.0)	0.8 (0.3-1.0)	0.58
VSS (range)	4 (1-9)	3 (0-6)	0.146
VAS (range)	7.6 (0.6-10)	7.1 (3.3-10)	0.681
